# Leukoencephalopathy hypomyelination with brainstem and spinal cord involvement and leg spasticity caused by *DARS1* mutations

**DOI:** 10.3389/fgene.2022.1009230

**Published:** 2023-01-12

**Authors:** Jingyi Zhu, Xiaomin Guo, Ningjing Ran, Jingtao Liang, Fuyou Liu, Junyan Liu, Rongyu Wang, Lianyan Jiang, Dongdong Yang, Meijun Liu

**Affiliations:** ^1^ Neurology Department, Hospital of Chengdu University of Traditional Chinese Medicine, Chengdu, China; ^2^ School of Clinical Medicine, Chengdu University of Traditional Chinese Medicine, Chengdu, China

**Keywords:** HBSL, aspartyl-tRNA synthetase, leukoencephalopathy, DARS, mutation

## Abstract

Hypomyelination with brainstem and spinal cord involvement and leg spasticity (HBSL), caused by aspartyl-tRNA synthetase (*DARS1*) gene mutations, is extremely rare, with only a few cases reported worldwide; thus, reports on HBSL treatment are few. In this review, we summarized the clinical manifestations, imaging features, treatment methods, and gene mutations responsible for HBSL based on relevant studies and cases.

## 1 Introduction

Hypomyelination with brain stem and spinal cord involvement and leg spasticity (HBSL, OMIM: 615281) is a leukodystrophy disease caused by a recessive missense mutation of the *DARS1* gene encoding cytoplasmic aspartyl-tRNA synthetase (AspRS). Leukodystrophies are diseases of brain white matter characterized by early onset, substantial mortality, and lack of treatment options. The population incidence is approximately 1 in 7,600 (Bonkowsky et al., 2010). HBSL onset usually occurs during infancy, and HBSL is characterized by motor impairment or stagnation of motor development, decrease in motor milestones, spasticity, ataxia, nystagmus, and even impairment in recognition (Taft et al., 2013; Wolf et al., 2015; Zhang et al., 2018; Min Tsui et al., 2020; Liu et al., 2022). The actual number of patients with HBSL is likely substantially higher than that reported (Muthiah et al., 2020).

Cytoplasmic aspartyl-tRNA synthetase (AspRS) charges aspartate-specific transfer ribonucleic acid (tRNA) with aspartate, which in turn catalyzes the aminoacylation reaction and promotes protein translation. AspRS belongs to a category of 36 aminoacyl-tRNA synthases (ARSs) that are critical for biological functions and are present in species ranging from bacteria to humans. These 36 ARSs lack functional redundancy (Antonellis & Green, 2008). All diseases caused by ARS deficiencies present with symptoms associated with the nervous system (Ognjenovic & Simonovic, 2018), which might be related to a decrease in the activity and change in the structure of key enzymes after the mutation of their respective genes. The cause of HBSL is consistent with these effects; however, the specific mechanism is unclear.

## 2 Clinical symptoms and neuroimaging features of HBSL

Our literature search identified 19 reported patients with HBSL (Taft et al., 2013; Wolf et al., 2015; Zhang et al., 2018; Min Tsui et al., 2020; Liu et al., 2022). Among them, one patient was from Australia, three were from China, two were from the United Kingdom, two were from the United States, and five were from India and Pakistan. Three patients with familial HBSL reported by Min Tsui et al., 2020 and three patients reported by Wolf et al. (2015) were of unknown origin; 11 patients were male, and 8 were female. The onset of HBSL usually occurs during infancy and early childhood. The age of onset in these reports ranged from 4 months to 18 years, with a mean age of 3.6 years. Nine patients were <1 year of age, five patients were 1–3.6 years, and five patients were >3.6 years old. Studies have reported that the earlier the onset of the disease, the poorer the prognosis. Among the reported infants (<3 years of age) with disease onset, most showed delayed motor development, associated with the characteristic HBSL symptoms of progressive leg spasticity, and the legs were heavier than the arms. The onset of the disease in older patients (≥3 years of age) was mostly motor impairment, gait instability, good recovery, and rare cumulative cognitive function. Motor milestones were also positively correlated with the age of onset. Moreover, 10 patients had abnormalities during pregnancy or delivery associated with a perinatal disease involving pregnancy, hypoxia during childbirth, or fetal position abnormalities. Furthermore, 16 patients had motor retardation, and seven patients had dyskinesia with nystagmus. Regarding their highest milestones, one patient could crawl, seven patients could walk, two patients could sit independently, two patients could stand alone, three patients could walk independently, and four patients could walk without support. Furthermore, six patients had cognitive dysfunction, five experienced deterioration in their condition due to pathological infection, and two had ictus epilepticus. On physical examination, 18 patients had hyperreflexia in the legs, 13 patients had positive Babinski signs, three patients had extrapyramidal signs, three patients had retinal abnormalities, one patient had a mild pale disc, one patient had a bilateral cherry red spot, one patient had pigment change, one patient had hypermetropia, one patient had optic atrophy, six patients had myopia, three patients had dysarthria, and five patients had ataxia (Supplementary Table S1). One patient had spinal cord tethering, one patient had vertebral malformations, one had trigonocephaly, one had Chiari malformations, one had a distal decrease in position and vibration sense of both legs, two had microcephalia, and one had dysdiadochokinesia.

The imaging features of HBSL mainly involved the supratentorial white matter, brainstem, cerebellum, spinal cord, corpus callosum, internal capsule, and periventricular region. T2-weighted imaging (T2WI) showed homogeneously high abnormal signals in the deep white matter and subcortical white matter, while T1-weighted imaging (T1WI) showed a slightly lower or equal signal. Furthermore, the internal capsule and corpus callosum were involved in 10 patients. Seven patients showed white matter thinning in the corpus callosum area without widespread atrophy in supratentorial white matter, 10 patients showed abnormal signals in the anterior brainstem, nine patients showed abnormal signals in the pyramidal tracts, one patient showed abnormal signs in the medial lemniscus, eight patients showed abnormal signs in the superior and inferior cerebellar peduncles without cerebellar atrophy, five patients showed abnormal signs in the white matter of the cerebellum, 12 patients showed abnormal signs in the spinal cord, nine patients showed abnormal signs in lateral corticospinal tracts, one patient showed involvement of the medulla oblongata, and two patients showed involvement of the periventricular region (Supplementary Table S1).

HBSL was first reported in 10 infantile-onset patients by Tafa in 2013. The cause was found to be missense mutations of the encoding cytoplasmic aspartate-tRNA synthase (AspRS) gene *DARS1* on chromosome 2q21.3, mostly in the C-terminal action-site domain of AspRS, which has highly conserved residues. This resulted in the non-synonymous substitution of the amino acids primarily located in and around the catalytic domain, which led to white matter disorders. HBSL is categorized as an autosomal recessive disorder (Taft et al., 2013). Genetic testing is the diagnostic method of HBSL; through whole-genome sequencing, whole-exome sequencing, and Sanger sequencing, the mutation sites and amino acid changes have been identified, which could support further research on leukodystrophies. Among the 19 cases reported, a total of 12 groups of gene mutations, three groups of homozygous mutations, nine groups of heterozygous mutations, and six groups of mutant gene loci were observed, while six groups were unknown. One group of homozygous mutations was reported in four patients (chr2: 136,680,399, c.766A>C, p.Met256Leu), one group of heterozygous mutations in three patients (c.536G>A, p.Arg179Lys) (c.1480C>G, p.Arg494Gly), one group of heterozygous mutations in two patients (chr2: 136,668,744, c.1379G>A,p.Arg460His) (chr2: 136,664,912,c.1480C>T,p.Arg494Gly), and one group of heterozygous mutations in two patients, (chr2: 136,668,733, c.1391C>T,p.Pro464Leu) (chr2: 136,664,912, c.1480C>T,p.Arg494Cys), along with one group of homozygous mutations in four patients previously mentioned and remaining two groups of homozygous mutations (chr2: 136,664,933,c.1459C>T, p.Arg487Cys) (c.1277T>C, p.Leu426Ser) (Table 1).

**TABLE 1:** DARS mutations associated with HBSL.

Subject	Country of origin	Genomic position (hg19)	cDNA mutation	Exon	Protein alteration	Inheritance	dbSNP ID
1	Australia	chr2: 136,673,803	c.1099G>T	11	p.Asp367Tyr	Paternal	-
		chr2: 136,678,161	c.821C>T	10	p.Ala274Val	Maternal	rs112205661
2,3,4,5, and 8	India and Pakistan	chr2: 136,680,399	c.766A>C	9	p.Met256Leu	Homozygous	-
4	India	chr2: 136,664,933	c.1459C>T	16	p.Arg487Cys	Homozygous	-
6,7	United States of America	chr2: 136,668,744	c.1379G>A	15	p.Arg460His	Paternal	-
		chr2: 136,664,912	c.1480C>T	16	p.Arg494Gly	Maternal	rs147077598
9,10	United Kingdom	chr2: 136,668,733	c.1391C>T	15	p.Pro464Leu	-	rs148806569
		chr2: 136,664,912	c.1480C>T	16	p.Arg494Cys	-	-
11	ND	-	c.599C>G	ND	p.Ser200Cys	-	-
		-	c.830C>T	ND	p.Ser277Phe	-	-
12	ND	-	c.1277T>C	ND	p.Leu426Ser	Homozygous	-
13	ND	-	c.839A>T	ND	p.His280Leu	-	-
		-	c.1099G>C	ND	p.Asp367His	-	-
14	China	-	c.1498_1499insTCA	ND	p.500_501insIle	-	-
		-	c.1210A>G	ND	p.Met404Val	-	-
15	China	-	c.1432A>G	ND	p.Met478Val	-	-
		-	c.1210A>G	ND	p.Met404Val	-	-
16,17, and 18	ND	-	c.536G>A	ND	p.Arg179Lys	Paternal	-
		-	c.1480C>G	ND	p.Arg494GLy	Maternal	-
19	China	chr2: 136,668,760	c.1363T>C	15	p.Y455H	Maternal	-
		chr2: 136,678,161	c.821C>G	10	p.A274G	Paternal	-

## 3 HBSL treatment

No consensus exists for HBSL treatment. Among the reported HBSL cases, only one patient died (Taft et al., 2013), one patient received immunoglobulin and steroid treatment, four patients received steroid therapy, two patients received interventions to improve muscle tone and rehabilitation exercise therapy, and one patient received neurotrophic substances to promote myelination regeneration. In the five patients treated with steroids, four patients showed improvements and two patients described improvements in motor abilities; however, the effects were short-term in one patient, who progressed several months later. While rehabilitation exercise therapy was also used to improve motor symptoms, they did not recover completely. The patients were mainly administered supportive and symptomatic treatment to improve their quality of life. Two patients who experienced viral infection and trauma, respectively, and showed improvement after analgesic or steroid therapy, could walk with support (Wolf et al., 2015), which was consistent with the overall treatment of leukodystrophy diseases. The prevention of infection, trauma, and stress in leukodystrophy is one of the treatment measures (Ashrafi & Tavasoli, 2017). In animal models, heterozygous mice with a normal phenotype had only 20% levels of AspRS protein compared to the levels in the control mice. Thus, increasing AspRS enzyme levels might be an effective treatment (Frohlich et al., 2017), and further studies are needed to improve other symptoms.

## 4 Discussion

HBSL is caused by missense mutations in the *DARS1* gene encoding the aspartyl-tRNA synthetase, conferring non-synonymous amino acid substitutions in highly conserved residues in the catalytic domain to cause white matter disorder (Taft et al., 2013; Wolf et al., 2015) (Min Tsui et al., 2020). These mutations occur due to changes at the active site or destabilization of side-chain interactions with the active site to impair aminoacylation (Taft et al., 2013; Muthiah et al., 2020). Cytosolic AspRS with other cytosolic ARSs and aminoacyl tRNA synthetase complex interact with multifunctional proteins (AIMPs) to form a multi-synthetase complex (MSC) that promotes translation by ferrying tRNAs to ribosomes (Lee et al., 2004; Khan et al., 2020). Thus, *DARS1* mutations might cause altered MSC function and play an essential role in HBSL. Taft et al. used a curated set of publicly available genomic data to investigate *DARS1* expression in humans and mice and found that mutant *DARS1* in HBSL was expressed across tissue types, including the central nervous system (Taft et al., 2013). *DARS1* is expressed in all organs, but in the adult brain, it is expressed mainly in the neuronal populations of the hippocampus, cerebellum, and cortex, with low expression in oligodendrocytes, astrocytes, and microglia present in the axons and dendrites of neurons that promote the synthesis of local proteins (Frohlich et al., 2018). Nerve cells are highly susceptible to protein translation disorders, and ARS gene mutations in the cytoplasm and mitochondria mainly cause central and peripheral lesions (Wallen & Antonellis, 2013). Among them, *DARS1* mRNA and protein are the most expressed in the cerebellum and are responsible for controlling motor functions (Frohlich et al., 2018). This area is susceptible to protein disorders; thus, *DARS1* mutations lead to motor dysfunction, progressive spasticity in the legs, and other cerebellar dysfunctions. Oligodendrocytes play a major role in myelination; low *DARS1* expression in oligodendrocytes makes them particularly susceptible to disturbances in protein synthesis caused by gene mutations. The functions of neurons and oligodendrocytes are closely linked to the central nervous system (Saab & Nave, 2017), and the activity of neurons can regulate oligodendrocyte differentiation and myelination (Gibson et al., 2014; Frohlich et al., 2017). Therefore, *DARS1* dysfunction in neurons can lead to the secondary impairment of oligodendrocyte function, ultimately leading to hypomyelination in HBSL (Frohlich et al., 2018). However, recent studies have shown that the primary pathological changes of HBSL are in axons rather than oligodendrocytes associated with myelin production. Studies in mice have also shown that *DARS1* is highly expressed in axons and synapses (Frohlich et al., 2017). Studies on LBSL found that myelin cell expression was not affected in *DARS2* cKO mice (Yamashita et al., 2013); however, evidence for HBSL is insufficient. *DARS1* mutations result in non-synonymous amino-acid substitutions, followed by the accumulation of non-functional, unfolded, or misfolded proteins of the endoplasmic reticulum, which triggers the apoptosis and clearance of abnormal cells (Lin & Popko, 2009).

Neurologic imaging is a primary supplementary examination for HBSL. In 19 patients with HBSL, the imaging features were highly consistent with the clinical presentation. The neuroimaging characteristics included diffuse, homogeneous, and symmetrical T2WI high signals of the supratentorial white matter, while T1WI showed variable intensity (hypointense, hyperintense, or isointense) depending on the degree of myelination of HBSL (Steenweg et al., 2010; Pouwels et al., 2014). However, some patients with later onset showed focal T2WI high signals. This observation was similar to that of other leukodystrophies, especially with leukoencephalopathy with brainstem and spinal cord involvement and lactate elevation (LBSL) (Ashrafi & Tavasoli, 2017). Both HBSL and LBSL involve the white matter of the brain, the dorsal columns, lateral corticospinal tracts in the spinal cord, either the pyramidal tracts or medial lemniscus in the brainstem, and the periventricular region. However, LBSL is caused by mutations in the encoding mitochondrial aspartate tRNA synthase gene *DARS2*. This differentiation (Muthiah et al., 2020) was confirmed by genetic analysis in patients 11 and 12, in whom LBSL was originally suspected, but HBSL was determined by Sanger sequencing (Wolf et al., 2015). Moreover, HBSL is also easily confused with conditions such as multiple sclerosis (MS), when some patients present focal changes in the spinal cord. Abnormal supra-white matter signals and the involvement of the internal capsule, callosum, and spinal cord are consistent with upper motor neuron (UNM) signs such as spasticity, hyperreflexia, and a positive Babinski sign. Spinal cord involvement is common in HBSL and is one of its characteristic manifestations. Magnetic resonance imaging examinations show abnormal long-segment signals of the spinal cord, distinguished from inflammatory demyelinating lesions with focal lesions (Filippi et al., 2019). Three patients with familial HBSL were reported in 2020. Their clinical symptoms were more severe than MRI monetization and might be associated with worsening of the condition due to viral infection, vaccination, or trauma.

The pathological mechanism of HBSL is unclear. Frohlich et al. (2017, 2020) constructed animal models of HBSL, which were difficult to develop and showed low survival with tardive dyskinesia, vacuolization and demyelination of spinal white matter, reduced size, body weight, fat mass, and microphthalmia. Hydrocephalus and anxiety were observed along with the aforementioned characteristics with the introduction of heterozygous *DARS1* ^*M256/–*^descendants, although body weight and fat mass reduction and microphthalmia were not reported (Klugmann et al., 2022). This study provided a basis for HBSL treatment, consistent with the condition of patient no.17 (Supplementary Table S1), who had received a ventriculoperitoneal shunt to reduce pressure due to hydrocephalus. Additionally, the irritability in patients 1, 7, 8, and 19 (Supplementary Table S1) requires further investigation of mental symptoms, which are also manifestations of white matter diseases. An effective treatment for HBSL is lacking; while steroids (Taft et al., 2013), immunoglobulins (Taft et al., 2013), neurotrophic drugs (Liu et al., 2022), and sports rehabilitation (Zhang et al., 2018) have been used for treatment, only some patients showed partial remission of their clinical manifestations and the course of HBSL development was not reversed. Although the short-term effects of steroid and immunoglobulin therapy in a few patients were similar to those of the inflammatory demyelinating disease, genetic and immune tests were not performed (Wolf et al., 2015). Previous studies have shown that it might be associated with IgM Fc receptors expressed in oligodendrocytes, oligodendrocyte precursor cells, and myelin sheaths, which promote the direct interaction of immunoglobulins with oligodendrocytes. They induce calcium inflow within oligodendrocytes and astrocytes, improve the inherent repair capacity of cells, and promote myelin regeneration (Nakahara et al., 2003; Paz Soldán et al., 2003; Stangel & Bernard, 2003; Trebst & Stangel, 2006). Additionally, repairing myelination through vegetative nerve methods can improve motor symptoms (Liu et al., 2022). While an effective method of HBSL treatment is generally lacking, promoting oligodendrocyte expression, reducing oligodendrocyte apoptosis, promoting myelination repair and regeneration, and targeting *DARS1* mutants can be used to develop effective treatment methods.
