# Supplementary material for: Leukoencephalopathy hypomyelination with brainstem and spinal cord involvement and leg spasticity caused by DARS1 mutations
Source: Front Genet. 2023 Jan 12;13:1009230. doi: 10.3389/fgene.2022.1009230 (PMC9878823; doi:10.3389/fgene.2022.1009230)
Supplement: Supplementary file 1 [file Table1.DOCX]

**Table 1: Basic information, clinical manifestations and neuroimaging features.**

| Patient no. | 1 | 2 | 3 | 4 | 5 | 6 | 7 | 8 | 9 | 10 | 11 | 12 | 13 | 14 | 15 | 16 | 17 | 18 | 19 |
| --- | --- | --- | --- | --- | --- | --- | --- | --- | --- | --- | --- | --- | --- | --- | --- | --- | --- | --- | --- |
| Gender | m | m | m | m | m | f | f | m | m | f | m | m | f | f | f | f | m | f | m |
| Age at presentation /Age of onset | 11m | 9m | 7m | 4m | 6m | 9m | 12-14m | 9m | 6m | 6m | 14m | 18y | 16y | 26m | 8y8m | 3y | 4 y 3 m | 7 y | 2y |
| Siblings (affected/unaffected) | No | 1 affected | 1 affected | 1 unaffected | No | 1 affected ,1 unaffected | 1affected,1 un affected | No | 1 affected | 1 affected | Unknown | Unknown | Unknown | No | No | 2 affected | 2 affected | 2affectedd | Unknown |
| Pregnancy/delivery/perinatal period | spina bifida occulta, tethered cord, solitary kidney | Normal | Normal | elective Caesarean section | normal | mild hyperbilirubinemia | mild hyperbilirubinemia, episode of apnea, gastroeosophag reflux | vacuum-assisted delivery | elective caesearean section | elective caesearean section | Unknown | Unknown | Unknown | Premature birth, suspicious hypoxia at birth | Normal | Maternal hypertension, vertebral deformities. | Maternal hypertension, | Maternal hypertension, | Normal |
| Unsupported walking (years) | never | never | never | never | never | never | never | never | never | never | Unknown | Unknown | 14m | Unknown | No， posture is abnormal and swinging. | 21 months to present (12 years) | 2 years old to present (1 1 years); clumsy, frequent falls | 9 months to present (14 years); occasional falls | never |
| **Presentation** | |  |  |  |  |  |  |  |  |  |  |  |  |  |  |  |  |  |  |
| Signs at presentation | axial hypotonia, hypertonia legs>>arm, irritable | spastic diplegia, squint, nystagmus, head bobbing | ankle tightness, nystagmus, head bobbing | delayed motor development, muscle hypotonia | delayed motor development, nystagmus | abrupt loss in motor milestones after vaccination | gradual loss of motor milestones | irritable, delayed motor development | spastic diplegia | spastic diplegia | make slow progress and learned to walk with support | lost the ability to walk without support legs>>arms | leg spasticity and urinary urgency and frequency | can't sit or stand alone, hands can actively grasp objects | delayed motor development | Brisk reflexes, positive Babinksi bilaterally, ankle clonus, short wide neck, capillary naevi | Brisk reflexes, positive Babinski bilaterally | Normal reflexes, positive Babinski bilaterally, | unable to stand up, faced difficulty in raising upper limbs |
| ***Course over time*** | |  |  |  |  |  |  |  |  |  |  |  |  |  |  |  |  |  |  |
| Motor | early normal, progressive spasticity in legs in course over time | early normal, progressive spasticity in legs in course over time | early normal, progressive spasticity in legs in course over time | early normal, progressive spasticity in legs in course over time | early developmental delay, progressive spasticity in legs in course over time | early normal, progressive spasticity in legs in course over time | early normal, progressive spasticity in legs in course over time | early normal, progressive spasticity in legs in course over time | early developmental delay, progressive spasticity in legs in course over time | early developmental delay, progressive spasticity in legs in course over time | Stagnation of motor development | | | early developmental delay | early developmental delay | | |  | Difficulty in walking, abnormal posture, and difficulty in standing, walk with support |
| Regression | transient mild regression during infections | No | No | No | No | second event of regression at 12 months after urinary tract infection | slow regression after 14 months | Yes | No | No | No | No | No | Drinking water is easy to choke, the articulation is not clear | No | No | Worsening gross and fine motor skills post-operative cranio-cervical decompression and meningitis | No | No |
| Cognition | early normal, mild mental retardation in course over time | mild mental retardation | mild mental retardation | mild mental retardation | normal | normal | normal | normal | normal | normal | above average | Unknown | Unknown | normal | Unknown | Mild learning difficulties | Learning difficulties | Normal | disorder |
| Highest motor milestone | stands and cruises | walks a few steps with support, diplegic gait | sits without support | walks with support, unstable gait | sits without support | stands and cruises | crawls and cruises | move with wheel chair | move with wheel chair | propel a supportive walker | Unknown | Unknown | Unknown | Unknown | Unknown | Independent walking | Independent walking | Independent walking | |
| Epilepsy | No | No | not done | Yes | No | No | No | No | Yes | No | not done | not done | not done | not done | not done | No | No | No | not done |
| Peripheral neuropathy | NCV normal | NCV normal | no | NCV normal | no | NCV normal | no | no | no | no | Not done | Not done | Not done | Not done | Not done | Not done | Not done | Not done | the right dorsal inter bone muscle and bilateral medial femoral myogenic damage |
| Treament |  |  |  |  |  | reported improvement with IVIG and steroids | reported improvement with steroids | |  |  | no improvement with steroids | reported improvement with steroids | reported improvement with steroids | Give symptomatic treatment such as improving muscle tone and rehabilitation exercise | Give symptomatic treatment such as improving muscle tone and rehabilitation exercise | | | | |
| ***Physical examination*** | |  |  |  |  |  |  |  |  |  |  |  |  |  |  |  |  |  |  |
| Head circumference | normal | small(<2SD) | normal | normal | normal | small(<2SD) | normal | small(<2SD) | normal |  | Unknow | Unknow | Unknow | small(45cm) | Normal | Betwee n -2 and -1 SD | normal | normal | Unknow |
| Height | normal | normal | normal | normal | normal | normal | normal | normal | normal | normal | Unknow | Unknow | Unknow | Unknow | Unknow | Normal | Normal | Normal | Unknow |
| Retinal abnormalities | mild optic disc pallor | nomal | nomal | nomal | bilateral cherry red spot | nomal | not done | pigmentary changes | optic disc pallor | optic disc pallor | Unknow | Unknow | Unknow | Unknow | Unknow | Unknow | Unknow | Unknow | Unknow |
| Vision | normal | hypermetropia | normal | decreased, optic atrophy | normal | normal | normal | myopia | myopia | myopia | Unknow | Unknow | Unknow | Unknow | Unknow | myopia | myopia | myopia | Unknow |
| Extraocular eye movements | normal | nystagmus | nystagmus | nystagmus | nystagmus | normal | normal | nystagmus | nystagmus | nystagmus | Unknow | Unknow | Unknow | Unknow | Unknow | Unknow | Unknow | Unknow | Unknow |
| Hearing | normal | normal | normal | normal | normal | normal | normal | normal | normal | normal | Unknow | Unknow | Unknow | Unknow | Unknow | Unknow | Unknow | Unknow | Unknow |
| Receptive language | normal | normal | normal | normal | normal | normal | normal | normal | normal | normal | Unknow | Unknow | Unknow | Unknow | Unknow | Unknow | Unknow | Unknow | Unknow |
| Dysarthria | yes | yes | no | no | no | no | no | no | no | no | Unknow | Unknow | Unknow | Yes | Unknow | Unknow | Unknow | Unknow | Unknow |
| Dysphagia, tube feeding | no | no | no | no | no | no | no | no | no | no | Unknow | Unknow | Unknow | Drinking water is easy to choke | Unknow | Unknow | Unknow | Unknow | Unknow |
| Axial tone | decreased | normal | normal | decreased | decreased | decreased | decreased | decreased | decreased | decreased | Unknow | Unknow | Unknow | Unknow | Unknow | Normal | Normal | Normal | Unknow |
| ***Arms*** | |  |  |  |  |  |  |  |  |  |  |  |  | Unknow | Unknow | Unknow | Unknow | Unknow | Unknow |
| Spasticity | no | mild | mild | mild | mild | mild | mild | no | mild | mild | Unknow | Unknow | Unknow | mild | mild | No | No | No | mild |
| Reflexes | normal | brisk | brisk | brisk | brisk | brisk | brisk | normal | brisk | brisk | Unknow | Unknow | Unknow | Normal | Normal | Normal | Normal | Brisk | Brisk |
| Ataxia | no | yes | no | mild | could not be assessed | no | no | no | yes | mild | Unknow | yes | Unknow | Yes | Yes | Yes | Yes | Yes | Yes |
| Extrapyramidal signs | no | no | no | no | no | dystonic posturing when reaching for objects | no | no | no | no | Unknow | Unknow | Unknow | No | No | No | No | No | Yes |
| ***Legs*** | |  |  |  |  |  |  |  |  |  |  |  |  |  |  |  |  |  |  |
| Spasticity | severe | severe | severe | severe | severe | severe | severe | severe | severe | severe | severe | severe | Unknow | None | None | Yes | None | None | No |
| Reflexes | Brisk | Brisk | Brisk | Brisk | Brisk | Brisk | Brisk | Brisk | Brisk | Brisk | Brisk | Brisk | Unknow | Brisk | Brisk | Brisk | Brisk | Brisk | Brisk |
| Babinski | positive | positive | positive | positive | positive | positive | positive | positive | positive | positive | Unknow | Unknow | Unknow | positive | positive | Unknow | Unknow | Unknow | positive |
| Extrapyramidal signs | No | No | No | No | No | No | No | No | No | No | Unknow | Unknow | Unknow | Yes | Yes | No | No | No | Yes |
| Plantar reflex | |  |  |  |  |  |  |  |  |  |  | distal decrease in position and vibration sense of both legs | | | | Unsteady on tandem gait | Unsteady on tandem gait | Unsteady on tandem gait | Unknow |
| **MRI** |  |  |  |  |  |  |  |  |  |  |  |  |  |  |  |  |  |  |  |
| Age at MRI | 3y | 5y |  | 8y | 4y | 6y | 2y | 2y | 8y | 20m | 2y | 19y | 18y | 16m | 3y | 3y | 5y | 7y | 45y |
| ***Signal of supratentorial white matter*** | |  |  |  |  |  |  |  |  |  |  |  |  |  |  |  |  |  |  |
| Homogeneously abnormal | + | + |  | + | + | + | + | + | + | + | + | + | + | + | + | + | + | + | + |
| Consistent with hypomyelination | - | - |  | + | + | + | + | + | + | - |  |  |  |  | - | + | + | + | + |
| ***Supratentorial atrophy*** | - | - |  | - | - | - | - | - | - | - |  |  |  |  |  | - | - | - |  |
| internal capsule | 11 | 11 |  | + | + | + | 11 | + | + | 11 |  |  |  | + | - | - | - | - |  |
| Corpus callosum | + |  |  |  |  |  |  |  |  |  |  |  |  |  |  |  |  |  |  |
| Thinning | + | + |  | + | + | + | - | + | + | - |  |  |  |  |  |  |  |  |  |
| ***Brainstem*** | |  |  |  |  |  |  |  |  |  |  |  |  |  |  |  |  |  |  |
| Abnormal signal of anterior brainstem | + | + |  | + | + | faint | faint | faint | faint | faint |  |  |  |  |  | - | - | - |  |
| pyramidal tracts | + | + |  | + | + | + | faint | - | faint | faint |  |  |  |  |  |  |  |  |  |
| medial lemniscus | - | - |  | + | - | - | - | - | - | - |  |  |  |  |  | - | - | - |  |
| ***Cerebellum*** | |  |  |  |  |  |  |  |  |  |  |  |  |  |  |  |  |  |  |
| Cerebellar atrophy | - | - |  | - | - | - | - | - | - | - |  |  |  |  |  |  |  |  |  |
| white matter | - | - |  | + | 111 | - | - | 1111 | 111 | - | + |  | - |  |  |  |  |  |  |
| superior cerebellar peduncles | + | + |  | + | + | + | + | + | + | + |  |  |  |  |  |  |  |  |  |
| inferior cerebellar peduncles | + | + |  | + | + | - | - | + | + | + |  |  |  |  |  |  |  |  |  |
| ***Spinal cord*** | |  |  |  |  |  |  |  |  |  |  |  |  |  |  |  |  |  |  |
| dorsal columns | + | + |  | + | + | faint | + | + | not done | not done |  | + | + | - | - | Yes from C1 to C6 | Yes from C2 to C7 | Yes C1 to C6 | + |
| lateral corticospinal tracts | + | not done |  | + | + | not done | + | + | not done | not done |  | + | + | not done | not done | Yes C1 to C6 | Yes from C2 to C7 | - | - |
| ***Proton MRS*** | |  |  |  |  |  |  |  |  |  |  |  |  |  |  |  |  |  |  |
| lactate elevated | not done | not done |  | - | not done | - | not done | - | not done | not done |  |  |  |  |  | Normal. mild elevation of choline and NAA compared to creatine | Normal. NAA:Cr ratio 1 .59 | Normal. But reduction in the normal NAA:Cr ratio 1 .28. | |
| medulla oblongata | |  |  |  |  |  |  |  |  |  |  | + |  |  |  |  |  |  |  |
| periventricular region | |  |  |  |  |  |  |  |  |  |  | + | + |  |  |  |  |  |  |
| **other** |  |  |  |  |  |  |  |  |  |  |  |  |  |  |  | Trigonocephaly, Slight in-toeing gait bilaterally, mild lumbar lordosis | Mild trigonocephaly, Chiari 1 with craniocer vical stenosis, craniocervical decompression (8 years old), post meningitic hydrocephalus with right sided VP shunt (8 years old), slight in-toeing gait bilaterally with mild lumbar lordosis. | Trigonocephaly, No in-toeing, mild lumbar | |
